# Patients' and health professionals' perspectives regarding shared decision making in the psychiatric inpatient setting – A multiple qualitative case study

**DOI:** 10.1016/j.pecinn.2024.100352

**Published:** 2024-10-21

**Authors:** Caroline Gurtner, Jos M.G.A. Schols, Christa Lohrmann, Sabine Hahn

**Affiliations:** a Applied Research & Development in Nursing, Department of Health Professions, Bern University of Applied Sciences, Bern, Switzerland; b Department of Health Services Research, Care and Public Health Research Institute (CAPHRI), Maastricht University, Maastricht, the Netherlands; c Department of Family Medicine & Care and Public Health Research Institute (CAPHRI), Maastricht University, Maastricht, the Netherlands; d Institute of Nursing Science, Medical University Graz, Graz, Austria

**Keywords:** Shared decision-making, Mental health services, Psychiatric inpatient setting, Interprofessional collaboration, Case study

## Abstract

**Objective:**

Shared decision-making is one promising approach to promoting recovery and person-centred care but seems challenging for implementation in clinical practice. This study aimed to explore how patients and health professionals experience shared decision-making and its facilitators and barriers.

**Methods:**

A multiple qualitative case study design was chosen, using a constant comparative method. Multiple data sources were used, including individual interviews, observation, document analysis and a focus group.

**Results:**

Through first a within-case analysis and then second a cross-case analysis, four patient profiles and their potential for shared decision-making were constructed. The results indicate that in the daily routine of the psychiatric inpatient setting different forms of decision making are used, even though health professionals advocate shared decision-making as the favored approach. Patients also have varying expectations and perceptions regarding shared decision-making, which is reflected in the degree of their involvement.

**Conclusion:**

Shared decision-making could be enhanced in the future by a more proactive communication style and the proactive provision of information on the part of health professionals, in order to enhance patient participation in decision-making.

**Innovation:**

The study identified different forms of decision-making within the acute psychiatric inpatient setting, highlighting the gap between the advocated SDM approach and its practical implementation. This divergence is a key aspect, as it underlines the complexity of implementing SDM in real clinical settings.

## Highlights

• The study highlights a gap between the advocacy of SDM and its practical implementation.
• Patients' perceptions regarding SDM differ, which is illustrated in typical patient profiles.
• SDM could be promoted by proactive communication, addressing patient's needs.
• Provision of information to patients is crucial to enhance SDM in clinical practice.

## 1 Introduction

Mental illnesses are relatively common worldwide and have an impact on various areas of the life of those individuals affected, for example through loss of employment, limited social networks, financial disadvantages and stigma [1]. In order to provide high quality mental health services, which are tailored to the needs of the service users, they should follow the principles of person-centred care (PCC) and focusing on recovery outcomes [1,2]. The recovery approach puts the persons with their individual health problems at the centre and encourages them to make self-determined decisions about their health and their future lives [3]. In this context, shared decision-making (SDM) is one promising approach to promoting PCC and the recovery orientation in clinical settings, showing positive effects on satisfaction with care received, reduces unmet needs, and subsequently might also reduce health-related costs in the long term [[4], [5], [6]].

SDM is described as a process in which at least two parties, usually the patient and a physician, engage in a joint discussion about the advantages and disadvantages of, for example, a specific treatment and come to a shared decision based on both, the clinical evidence and the patient's preferences [7,8]. In clinical psychiatric practice, three approaches have been discussed in the literature regarding the different styles of decision-making used: a passive, a shared and an active (or informed) decision-making style [6,9,10]. Out of these three, the shared decision-making style is described as the “*optimal way”* to promote patient involvement and to improve the quality of care [6,11,12]. However, implementing SDM in clinical practice seems complex and challenging [5]. For example, the heterogeneity of patient situations, structural preconditions such as the professionals' understanding of their roles as well as routine procedures represent a particular challenge. As for patients, insufficient information, or the fear of negative consequences by engaging in decision making represent further barriers to SDM [13,14].

Theoretical models focusing on the implementation of SDM in health care settings have gradually expanded over the last decades. In addition to communication aspects, these models also address organisational factors such as the institutional culture, the attitudes of health professionals, as well as work-related processes and workflows within a particular setting [[13], [14], [15], [16], [17]]. On an operational level, the process of SDM in the psychiatric setting has been illustrated by Gurtner et al. [18] and emphasizes a trusting relationship among the patient and the health professionals involved. While the conceptual understanding of SDM in mental health is still inconsistent, more recent research point out the need to involve a broader range of stakeholders within the SDM process, such as family members and peer workers [[19], [20], [21]].

Regarding the implementation of SDM in clinical psychiatric practice, various challenges and barriers are encountered within this setting. First, the needs for treatment as well as the treatment goals, which are identified by the patient on the one hand, but also by the health professionals on the other, can differ fundamentally [22]. Second, the preconditions for SDM also depend on whether the patient is undergoing treatment voluntarily or involuntarily, because involuntary treatment negatively affects the therapeutic relationship [23]. Involuntary admissions to psychiatric hospitals have increased in several European countries over the last 20 years, affecting one in two patients in the UK and one in five patients in Swiss, Austrian or German psychiatric hospitals [[24], [25], [26], [27], [28]]. Finally, patients and health professionals have different understandings of the meaning of SDM [5].

What this all points to is a need for further exploring enablers and barriers in the psychiatric inpatient setting from the perspective of all stakeholders, on how SDM can be promoted and implemented into everyday practice [13,21,29]. The theoretical discourse to date also reflects the emphasis on research within the different mental health care settings, as the theoretical concepts of SDM do not fully reflect their challenges and specifics [21,30]. Furthermore, data on the involvement in decision-making of patients in need of acute psychiatric care and their role in the SDM process are, thus far, inconclusive [13,31].

The aim of this study was to explore the individual experience of SDM from the perspective of patients in the acute psychiatric inpatient setting. Furthermore, the study sought to investigate whether patient perspectives regarding SDM were in alignment with those of the health professionals.

## 2 Methods

### 2.1 Design

A multiple qualitative case study design was chosen as a general research strategy. The underlying theoretical propositions, as suggested by Yin [32], integrated the findings of a previous consolidative literature review on the conceptual understanding of SDM in psychiatric care [18]. These indicate that SDM depends on, among other things, the understanding of the role that individuals involved in the decision-making process assign to themselves as well as their individual expectations and preferences for decision-making [15,16]. This study provides an initial in-depth exploration of the patients and health professionals perspective regarding SDM in the psychiatric inpatient setting. The results should be regarded as preliminary and can be extended within other settings [33,34].

Case studies aim to examine the phenomenon under investigation in a real-life context [32,35,36]. Regarding the present study, it was assumed that the context and structural factors of the psychiatric inpatient setting have an influence on the behaviour of the persons participating in the SDM process. For this purpose, an explorative and descriptive approach was chosen [32]. To obtain a comprehensive insight into the experience of SDM from the perspective of patients and health professionals, various data sources were used, including individual interviews, observation, document analysis and a focus group [[37], [38]].

A purposive sampling strategy was applied, and potentially participating patients were selected based on the type of criterion sampling [33]. The criteria for the sample were therefore that the patients should be representative of what would be expected in an acute psychiatric inpatient ward, and vary in terms of age, diagnosis, voluntary admission and experience in the psychiatric setting. The health professionals were selected using expert sampling. In particular, their focus on patient-centred care and preference for SDM was an important characteristic of the inpatient ward and the reason for conducting the study there [34]. Regarding the purposeful sample, data saturation in this qualitative case study was related based on the richness of the data, which were collected for each case using different methodological approaches and thus allowed to integrate diverse perspectives on each case in the sense of data triangulation [35,36,39]. This means that several different units of data were synthesised into one case, which in our study was defined as a patient case.

### 2.2 Participants

On predefined data collection dates, the patients were selected purposively by an internal study coordinator and approached by the ward manager with the aim of obtaining a variety of patient profiles in need of acute inpatient treatment. The patient situations were to differ in gender, age, diagnoses, length of hospital stay and whether they had been voluntarily or involuntarily admitted. The health professionals working on the selected ward and participating in this study were to represent the interprofessional core team consisting of two nurses, one psychologist and one physician. All of them were involved in the treatment of the patients participating in this study.

### 2.3 Data collection

Due to restricted accessibility in Swiss hospitals because of COVID during the data collection period between February and June 2020, a selection of study dates had to be predefined with the ward management. The study was presented before and after data collection to an internal research board of the selected psychiatric hospital and all steps were organized and discussed with an internal study coordinator, who supported data collection.

**Fig. 1:**
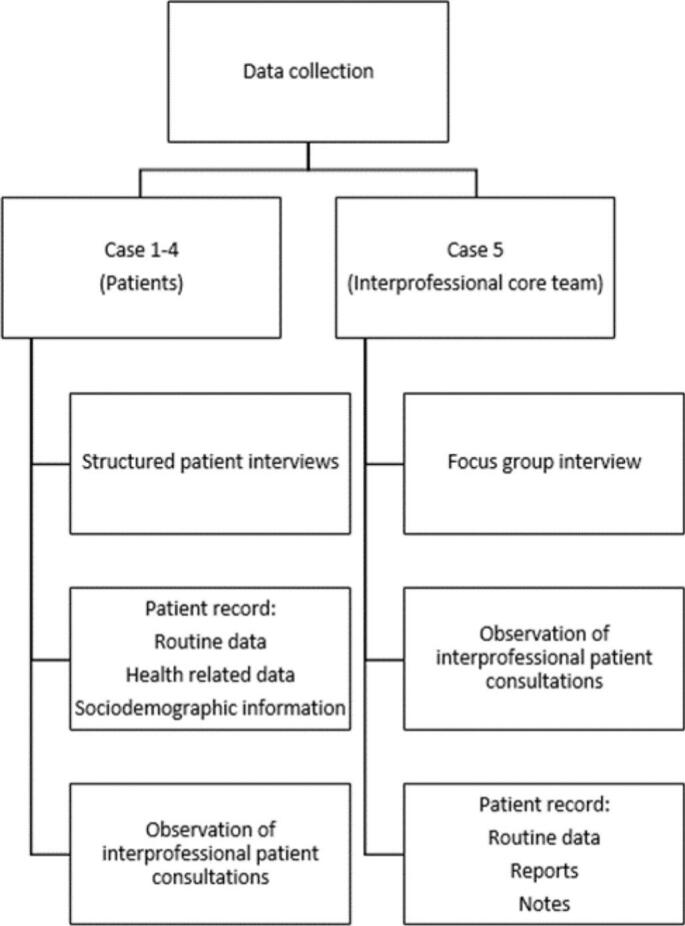
Overview of data collection per case.

Data collection included multiple data sources: interviews with the patients, a focus group with the interprofessional core team and observations of interprofessional patient consultations (including both the perspective of the patient and the interprofessional core team). Routine data from patient assessments during admission as well as reports and notes from the perspective of the interprofessional core team were obtained from the patient record (Fig. 1).

The one-to-one interviews with the patients and the observation of the interprofessional patient consultations took place in an office in the corresponding ward. The interviews were audio recorded, and field notes were taken during the observations. The interviews followed a semi-structured topic guide and aimed to gain an in-depth understanding of patients' perspectives on their health-related situation and SDM (Table 1).

**Table 1:** Topic guide for interviews with patients and focus group with health professionals.

Topic	Patients	Health professionals
Introduction: • Objective of the interviews	To gain a more in-depth understanding of how patients experience SDM	The role of the different health professionals in the decision-making process and their attitudes towards SDM
Main part:	Expectations towards and satisfaction with the care reived Description of an important decision about the treatment during the inpatient stay Patients' feelings and perception about their involvement in decision-making	Health professional's role in the team and in relation to SDM Understanding of SDM and its implementation in daily practice with patients Description of typical situations with SDM
Closing:	Inputs for improvement to feel (more) involved	Suggestions for change so that SDM is better implemented in the future

The focus group with representatives from the interprofessional core team was held on the morning after the individual patient consultations, when most of the patients had appointments or were outside the ward for a walk. The moderation of the focus group also followed a topic guide, which was comparable to that for patients but focused on the health professionals' perspective (Table 1).

The interviews, the focus group and the observations of the patient consultations were conducted by the first author, a trained nurse and nursing scientist with senior experience in the inpatient psychiatric setting and in qualitative research. An internal study coordinator supported the first author when extracting the data from the patient documentation. An overview of the data set collected for each case is provided in Fig. 1:

### 2.4 Ethical consideration

Patients and health professionals were selected and approached with the request to participate in the present study by the ward manager, who was not part of the interprofessional care team. All participants (patients and health care professionals) were informed verbally and in writing, that participation in the study was voluntary and could be withdrawn at any time. Furthermore, written consent was obtained prior to data collection. According to the Swiss national legislation, ethical approval in the form of a clarification of jurisdiction was obtained from the ethics committee of the Canton Bern (January 2020), which decided that the study did not fall under the Swiss National Federal Act on research involving human beings (BASEC number: Req-2020-00081).

### 2.5 Data analysis

The individual interviews with the patients and focus group with the interprofessional core team were transcribed verbatim and translated from Swiss German to High German prior to data analysis. Subsequently, the data obtained from multiple sources were then transferred to MAXQDA 2022 (VERBI Software, 2021) to organise and analyse the data.

The analysis of the data was carried out in an iterative way, including a number of steps, and followed a descriptive approach. For this purpose, we brought together the interview transcripts, the data from the patient records and the field notes from the observations on a case-by-case basis and consolidated them using a content structuring qualitative approach [40]. Therefore, segments relevant to the research questions and addressing the themes from the topic guide were labelled with an initial code, and in a further step, assigned to superordinate categories. Each case was analysed as an individual unit by the first author and checked by at least one of the co-authors independently. The co-authors have a background as health, medical and nursing scientists with senior experience in mental health care, qualitative research and case study design.

In a second step, a detailed and structured case description was written for each of the four patient cases (Case 1–4) and the core team (Case 5), addressing the experiences, perspectives and contextual factors of each case in relation to SDM. [32,40]. The case descriptions thus also formed the starting point for the individual patient profiles as part of the within-case analysis in the results section [32,33,40].

The last step comprised a cross-case analysis, which compared the perspectives of both, the patients and the interprofessional core team regarding SDM [32,[40], [41]]. Therefore, the constant comparative technique described by Boeije [42] was used. The subordinate categories from the first step and codes from the within-case analysis were charted using a Microsoft Excel (16) worksheet to compare them across cases [32]. During the cross-case analysis, the categories and codes were iteratively reviewed and specified by consulting the preliminary data (transcripts, field notes and data obtained from the patient records). In addition, the entire analysis process was continuously reflected upon and discussed with the team of co-authors. The authors followed the Consolidated Criteria for Reporting Qualitative Research (COREQ) for reporting the results of this study [43].

### 2.6 Rigor

The following four criteria proposed by Guba and Lincoln [44] were used in evaluating this study: credibility, transferability, dependability and confirmability. Credibility was addressed by member-checking the results by presenting them to representatives of the interprofessional core team and members of hospital management. Transferability of the results into settings other than acute psychiatric care was not intended because of the special interest in this context. To ensure dependability, the co-authors and experts from the psychiatric hospital were involved in reviewing the research plan, and multiple data sources with different data collection methods were used in the sense of data triangulation. Confirmability was addressed by acknowledging the first and last authors' predispositions as a trained nurse and as a researcher with experience in a comparable setting.

## 3 Results

The results of this qualitative case study are presented within three main sections. The first part comprises a) a description of the health-related characteristics of the participating patients, obtained from the patient record, b) the characteristics of the participating health professionals representing the interprofessional core team and c) the cultural context of the study site with relation to SDM. The second part presents the results of the within case analysis and includes four exploratory case descriptions that reflect the individual experiences of each patient in relation to SDM. Finally, the third part comprises the themes of the cross-case analysis, exploring the patients' and the health professionals' understanding and application of SDM in the psychiatric inpatient setting.

### 3.1 Participants and context

#### 3.1.1 Health related characteristics of the participating patients

Three of the four patients who took part in the study were female, and all of them were White in terms of their race. Their age ranged from 20 to 71 years and none of them was married. All participating patients had more than one psychiatric diagnosis, two of them also had a somatic diagnosis (Table 2). The rating on the Health of the Nation Outcome Scale (HoNOS) obtained from the routine data in the patient record was used to further describe patient characteristics; it varied between a total score of 16–20 (max = 48). The HoNOS is an internationally used, validated instrument for the fine assessment of social functioning in clinical practice; it was used in the German Version [45].

**Table 2:** Characteristics of participating patients.

Case Number	Age (years)	Sex	Type of stay	Main psychiatric diagnose (ICD Code)
Case 1	20	female	transitional	panic and anxiety disorder (F.41.0)
Case 2	59	male	involuntary	depression and suicide attempt (F 33.2)
Case 3	23	female	voluntary	borderline personality disorder (F 60.31)
Case 4	71	female	voluntary	dependency disorder (alcohol) (F 10.2)

#### 3.1.2 Characteristics of the interprofessional core team

Four health professionals participated in the focus group interview and were represented as Case 5. All of them were members of the interprofessional core team and involved in the patient situations assessed. Two of the participants represented the group of nurses, one the group of psychologists and one the group of physicians. The demographics of the health professionals are presented in Table 3:

**Table 3:** Characteristics of participants of the focus group (interprofessional core team).

Case number	Age (years)	Sex	Professional Group	Years of experience
Case 5	47	female	nurse	>10y
Case 5	55	female	nurse	>10y
Case 5	29	female	physician	1-5y
Case 5	26	female	psychologist	1-5y

#### 3.1.3 Context

The psychiatric hospital selected was located in an urban area in the German-speaking part of Switzerland. Patients with all clinical psychiatric conditions could receive treatment there. In the hospital's mission statement, the management emphasised that the patient's personality and autonomy were important values and that treatment goals should, whenever possible, be defined in consultation with the patients.

The guiding concepts for the health professionals working on the acute psychiatric inpatient wards followed the principles of recovery and empowerment. Therefore, the patient and health professionals involved were encouraged to establish common treatment goals. Furthermore, provision of care and treatment by the health professionals was based on the notion that the health care receiver and provider formed a partnership of equals. In addition, the patient-related process in the selected ward was organized following a *core teamwork approach*. This meant an interprofessional core team was responsible for a patient's entire treatment process and planned and coordinated the treatment accordingly.

### 3.2 Within-case synthesis: Individual patient profiles

**Fig. 2:**
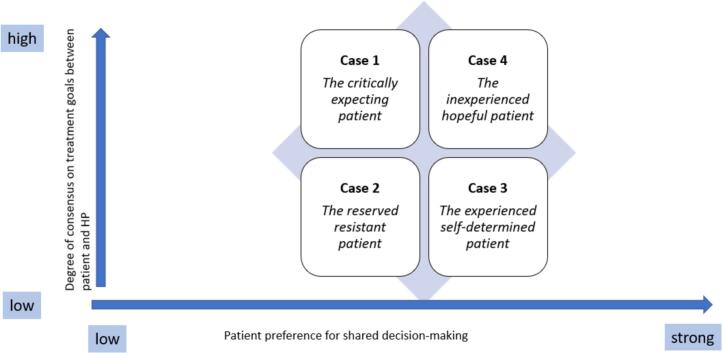
Patient profiles on a two-dimensional characteristics' framework.

As a result of the within case analysis, following a descriptive approach, the patient's individual characteristics and perspective on SDM in the psychiatric inpatient setting was abstracted for the presentation of the results. The following patient profiles were constructed (Fig. 2): The critically expecting patient (Case 1), the reserved resistant patient (Case 2), the experienced self-determined patient (Case 3) and the inexperienced hopeful patient (Case 4).

Each of the four profiles was assigned to a two-dimensional characteristic framework with reference to SDM, visualised in Fig. 2. The most important patient-related characteristics regarding SDM were the following: previous experience in the specific setting, patient's perception of the role of the health professionals in the decision-making process, patient's perception of their personal role in the decision-making process, personal goals regarding treatment and voluntary/involuntary hospital stay.

#### 3.2.1 The critically expecting patient

This type of patient expects health professionals to act proactively by providing information and support regarding diagnosis, treatment and therapy. She argues that the health professionals have therefore the necessary expertise, which she does not possess.

It's in my opinion not the patient's job to inform themselves, it's the health professionals' job. I simply expect for example, the diagnosis to be explained to me. (Case 1)

In terms of decision making, she generally has a rather passive view of her role. However, when it comes to making decisions that are particularly important to her, she behaves differently and clearly favours an active and self-determined role. As a consequence, this patient makes certain decisions without involving health professionals.

I ended up taking my vitamins myself at home because I assumed that they would either be forgotten or not dosed correctly. It was important to me that I could take them. (Case 1)

While basically agreeing with the treatment goals set by the health professionals and reporting an overall positive effect on her symptoms as a result, she is at the same time distrustful of health professionals' competencies due to a previous negative experience, where her medication dose was delivered incorrectly.

#### 3.2.2 The reserved, resistant patient

This type of patient has a completely different agenda than the health professionals. He has been admitted to the psychiatric hospital involuntarily because of a suicide attempt. He indicates that he wants to end his life again after his stay in the psychiatric hospital and therefore he does not see any sense in the treatment recommended.

All measures taken by the health professionals are unnecessary from my point of view, even though I can understand the intention behind them. (Case 2)

During the treatment consultation with the core team, he acts in a reserved manner and gives evasive answers to the questions raised by the health professionals. While he accepts the situation as unchangeable at the moment, he does not agree to therapy itself, which he sees as a waste of energy and time.

I see no reason why I should be given more information about the medication. I've told the doctors in no uncertain terms that I will attempt suicide again after being discharged from the hospital. (Case 2)

#### 3.2.3 The experienced self-determined patient

After long experience with psychiatric hospitals and her illness-related symptoms and treatment, this type of patient knows what works for her. She makes a strong claim to be involved in treatment decisions, but also deems herself an expert superior to the health professionals.

I made myself a specialist and I know more than the doctors do. (Case 3)

Knowing how it works in inpatient psychiatry, she adapts her behaviour so as to force through /her personal agenda. To attain her personal goals, she also ignores treatment-related recommendations made by the health professionals.

I will assert myself by any means, if I don't like something. (Case 3)

#### 3.2.4 The inexperienced hopeful patient

This type of patient has been admitted to a psychiatric inpatient setting for the first time and has no experience with the processes on the ward and the possibilities regarding treatment and therapy. Nevertheless, the personal agenda of this patient fits perfectly with the agenda of the health professionals, and she is hopeful that their common goals will be attained during the hospital stay. She declares that she has been admitted to hospital of her own free will, which on the one hand leads to a feeling of freedom of choice and autonomy. On the other hand, the lack of previous experience on an inpatient psychiatric ward is also reported as stressful and let to a feeling of disorientation.

Being hospitalized on a psychiatric inpatient ward feels like a a trip in the desert. (Case 4)

In particular, the lack of proactive communication about schedules and procedures was perceived as a barrier to decision making, as she had to spend a lot of time and effort to access the information, she needed to feel oriented.

There are many things that were not communicated to me. So, I had to explore the whole ward myself, and that's a weak point. (Case 4)

### 3.3 Cross case synthesis: Health professional and patient perspectives on how SDM works in clinical practice

#### 3.3.1 Different perceptions of SDM among health professionals and patients

The participating members of the interprofessional core team agreed that SDM should be practiced whenever possible. The concept of SDM was known from medical education and was practised with the idea that, as a first step, the patient was provided with a proposal for treatment. In a second step, if the patient disagreed or made a proposal himself/herself, the participants said they were open to discussing the decision further with the patient.

We (the interprofessional core team) make a recommendation, which we usually also discuss in advance. If the patient then wants something else, we are open to discussing alternatives with him. We don't stick to our point of view. (Case 5)

In comparison, participating patients' understanding of SDM was less consistent than the professionals', and how they experienced SDM depended strongly on their individual situation. Their perceived involvement in SDM was influenced either by the role in the decision-making process they ascribed to themselves, or they expected from the health professionals. Furthermore, it also depended on whether they had previous experience in the inpatient psychiatric setting, whether they were admitted voluntarily and whether they shared the same goals as the health professionals.

#### 3.3.2 Variations of decision-making practiced in the psychiatric inpatient setting

**Fig. 3:**
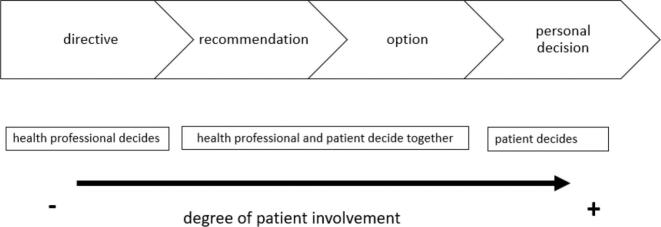
Different forms of decision making in the psychiatric inpatient setting.

Treatment decisions in the clinical setting were differentiated by the members of the interprofessional core team regarding their form (Fig. 3). They identified four different forms of decision-making used in the acute psychiatric inpatient setting which influence the degree of involvement of the patients. The forms were labelled as ‘directive’, ‘recommendation’, ‘option’ and ‘personal decision’ and are displayed below:

##### 3.3.2.1 ‘Directives’

In situations where patients have been admitted involuntarily, where patients refuse to acknowledge their illness or reject treatment, members of the interprofessional core team stated that they give ‘directives’ instead of options, and in this case, they do not expect any input from the patient:

I find it very difficult with the concept of SDM when we have patients who have been admitted involuntary, who do not show any understanding of their illness, and where the worlds then diverge completely. This is where you notice that the involvement of the patient is not realistic. (Case 5)

Depending on the individual profile of the patient, the reaction to a ‘directive’ about treatment had different effects. Two of the patients reported that receiving ‘directives’ led to either passive behaviour in decision-making, in which they pretended to agree, but personally had other plans (Case 2 and 3). This was also the case when patients reported that they understood the health professional's motivation behind the ‘directive’. Moreover, these thoughts and feelings were not actively communicated to the health professionals during the patient consultations but were expressed in the interviews.

I do not agree that I have to ask to leave the ward for a walk. But of course, I understand the reasons behind it. (Case 2)

##### 3.3.2.2 ‘Recommendations” or ‘options' as equivalent to SDM

Participating members of the interprofessional core team mentioned that whenever possible and when there was no acute risk situation, SDM should be practised. Therefore, patients were expected to become involved in decision making either by being given a pre-discussed ‘recommendation’ they can agree or disagree with or by being given ‘options’ they can choose from:

I have a patient right now for whom we have recommended Abilify (medication to treat major depressive disorder in adults). But he's not taking it now, he's taking Olanzapine (medication of the group of atypical neuroleptics), and he would also like to go up from 10 to 15 mg, which is fine with me if he prefers to take Olanzapine. So, it's better than if we prescribe Abilify and he doesn't take it. (Case 5)

For the patients interviewed, it was less important whether health professionals made a ‘recommendation’ or offered ‘options’ in order to be able to participate in treatment decisions. Thus, they expected the health care professionals to be proactive in providing the information needed and tended not to ask for it. In addition to the information available, two of the patients interviewed also explained their rather passive behaviour in the decision-making process in terms of their perception of the role of health professionals and their professional expertise. The critically expecting patient (Case 1) strongly felt it was the duty of the health professionals to inform the patient, while the inexperienced hopeful patient (Case 4) thought that competence in health-related issues clearly lay with the health professionals:

These are health professionals, and I can't just appear as a patient and say that I know better. Of course, I also have my competences in other fields and there I would say what I think. (Case 4)

##### 3.3.2.3 ‘Personal decisions’

There were also decisions where the members of the interprofessional core team did not expect themselves to be the ones who decided. This was the case when the decision was in their opinion of a personal nature, for example what form of housing to choose after the hospital stay or did not fall into their clinical expertise. This type of decision was labelled as a ‘personal decision’ and allowed the highest degree of patient involvement, because patients were encouraged to make the decision autonomously.

I think we always have to distinguish between decisions that are made by the patients, i.e. decisions that we think the patients should make on their own. (Case 5)

Making ‘personal decisions’ without involving the health professionals was in the patients understanding associated with taking over control when they considered a decision of importance to them and seeing themselves as ‘the expert’ due to considerable experience, for example in the setting, with the management of symptoms or treatment.

I was given an unknown dose of my medication. However, I simply took my usual dose. Otherwise, I would have simply refused to take the medication in the first place. (Case 1)

## 4 Discussion and conclusion

### 4.1 Discussion

The results of our multiple qualitative case study indicate that in the daily routine of the acute psychiatric inpatient setting different forms of decision making are used, even though health professionals clearly advocate SDM as the favored approach. Patients also have different expectations and perceptions regarding SDM, which is reflected in the degree of their involvement in decision making and illustrated on a two-dimensional characteristic framework. The involvement of the four patient cases in decision making referred to a spectrum ranging from passive to active behaviour. These findings might provide first insights into how to promote the practical developments of SDM in the psychiatric inpatient setting. However, there are underlying factors challenging SDM in this specific setting and these are, among other things, involuntary hospitalization, first admission to a psychiatric hospital and lack of information on treatment, but especially on the diagnosis and on medication.

The health professionals were committed to applying SDM in a dyadic manner with the patient and with at least two health professionals being present during a patient consultation. However, depending on the individual patient situation, they either used a more directive or more informed approach than SDM. The findings of this study support the theoretical debate regarding the use of different styles of decision making in clinical practice [6,9,10]. Besides, both the PCC approach and the recovery approach clearly advocate considering the patient's preferences and perceptions in decision making, however, those could hardly be considered by health professionals when using a passive decision-making style [13]. In addition, to potentially strengthen a person centered and recovery-oriented approach, the involvement of a polyadic consultation situation with family members or peer support workers could strengthen the patient's role in decision-making in the psychiatric inpatient setting and should therefore be promoted [21,46].

Furthermore, using a more directive decision-making style by giving orders may lead to a disadvantage of SDM for the group of patients who, for example, are excluded due to the lack of awareness of their illness or because of their involuntary hospitalization. Although the SDM approach involving this specific group of patients is widely advocated, its application poses an ethical dilemma, especially when it comes to decisions on medication [25]. Medical doctors on the one hand assume that the pressure exerted by an order instead of SDM will positively affect the patient's health status, while the SDM approach might have negative consequences for the patient [31]. Patients, on the other hand, perceive the legal context as a barrier to participating openly and honestly with their preferences in the decision-making process [13]. This supports a broader conceptual approach of SDM, which places a trustful relationship between the health professional and the patient at the center and considers both perspectives in the decision-making process equally [18].

Conflicting situations in daily clinical practice could make SDM nearly impossible [22]. This was true in our study, for example, concerning the reserved resistant patient (Case 2), where the treatment-related goals of the interprofessional core team and those of the patient diverged completely. However, to support the principles of PCC and recovery in the future, it would be particularly important to involve challenging patients in SDM, as this might improve the overall patient-related outcome [25,47].

This study's findings also showed that patients struggle with an insufficient level of information and spend a lot of time and energy orientating themselves in the unfamiliar environment of the acute inpatient psychiatry. These are in line with a study from Norway involving patients with a psychotic mental condition, which showed that most of them had received too little information on the current health situation as well as on treatment options, which prevented them from participating in SDM [48]. A more proactive and structured provision of information by health professionals could thus also be a simple and resource-saving approach to encourage patients to participate in SDM.

### 4.2 Innovation

The findings of this multiple qualitative case study contribute to the field of inpatient psychiatric care by providing new insights and practical implications on SDM. The study identified different forms of decision-making within the acute psychiatric inpatient setting, highlighting the gap between the advocated SDM approach and its practical implementation. This divergence is a key aspect, as it underlines the complexity of implementing SDM in real clinical settings. The existing models on SDM used, are limited in their ability to adapt to the complex dynamics of psychiatric care, for example when patients are hospitalized against their free will, requiring tailored specifications that prioritize the authentic perspective of the patients [21]. The study's recommendation for more proactive and structured information provision by health professionals addresses a further significant gap in patient care. This practical implication offers a simple yet effective strategy to promote SDM and is a necessary contribution to improving patient engagement. This includes extended solutions for information delivery during regular patient consultations, creating a space where questions and disagreement are not only tolerated but actively encouraged and discussed at every opportunity. One such innovative solution could be the implementation of polyadic consultations, where family members or peer support workers can be involved and strengthen the patient's role in decision making. Another practical solution to enhance SDM in a particular setting could be to train health professionals to recognize and adapt to the different decision-making preferences and needs of patients, moving fluidly between SDM, directive and informed approaches as appropriate.

### 4.3 Strengths and limitations

A strength of this study was the use of a qualitative case study approach. By collecting a variety of data from both the perspectives of the patients and the health professionals and by comparing them, the essence of typical patient situations in the context of the psychiatric inpatient setting could be explored. The findings can therefore serve as a powerful tool in the education of future health professionals by improving subsequent training programs, and as a catalyst for a deeper understanding of the individual strategies patients and health professionals use to navigate the challenging landscape of SDM.

One of the limitations of this approach is that qualitative case studies tend to focus on a single case or a small number of cases. They are also very context specific. As a result, the transferability of the findings to other contexts is limited and adaptations are needed to apply the findings to settings other than the inpatient psychiatry. Furthermore, the participating hospital had a strong focus on patient-centered care and SDM was a familiar concept. It can therefore be assumed that there was a certain degree of openness to approaches such as SDM, which also may have influenced the results.

## 5 Conclusion

This study has identified several facilitators and barriers to SDM in the acute psychiatric inpatient setting, based on the perspectives of both patients and the health professionals involved. Our results indicate that even when the health professionals' attitude and ward-related culture tended to be open to SDM and incorporated the principles of recovery and PCC in their internal guidelines; the realities of patients as well as of clinical practice do not always support SDM.

## Funding

This research did not receive any specific grant from funding agencies in the public commercial, or not-for-profit sectors.

## CRediT authorship contribution statement

**Caroline Gurtner:** Writing – original draft, Visualization, Software, Project administration, Methodology, Investigation, Formal analysis, Data curation, Conceptualization. **Jos M.G.A. Schols:** Writing – review & editing, Validation, Supervision, Methodology, Conceptualization. **Christa Lohrmann:** Writing – review & editing, Validation, Supervision, Methodology, Conceptualization. **Sabine Hahn:** Writing – review & editing, Validation, Supervision, Methodology, Conceptualization.

## Declaration of competing interest

The authors declare that they have no known competing financial interests or personal relationships that could have appeared to influence the work reported in this paper.

## Appendix A Supplementary data

Supplementary material. Image 1
